# Tyrosine Hydroxylase-Expressing Neurons in the Vagal Ganglia: Characterization and Implications

**DOI:** 10.3390/biomedicines13092126

**Published:** 2025-08-31

**Authors:** Artin Khaky, Nicole Lee Yang, Valerie van Weperen, Shail Avasthi, Neil Jani, Marmar Vaseghi

**Affiliations:** UCLA Cardiac Electrophysiology Programs, Division of Cardiology, Department of Medicine, University of California, Los Angeles, 100 Medical Plaza, Suite 660, Los Angeles, CA 90095, USA; artinkhaky@g.ucla.edu (A.K.); nicolely@g.ucla.edu (N.L.Y.); valerie.vanweperen@gmail.com (V.v.W.); savasthi@mednet.ucla.edu (S.A.); njani@mednet.ucla.edu (N.J.)

**Keywords:** parasympathetic, sympathetic, nodose, vagus, stellate, norepinephrine

## Abstract

**Background/Objectives:** A combination of sympathoexcitation and parasympathetic withdrawal contributes to the occurrence of ventricular arrhythmias, sudden cardiac death, and progression of heart failure after myocardial injury. As a result, vagal nerve stimulation has been under investigation as a potential option to increase cardiac vagal tone, but the results of clinical trials have been mixed. Prior studies have suggested that the vagal ganglia and nerves may contain sympathetic neurons that express tyrosine hydroxylase (TH), which, if stimulated, could potentially mitigate the effects of vagal nerve stimulation. The goal of the current study was to better characterize these neurons. **Methods:** Immunohistochemical staining was performed to evaluate for the presence of TH-expressing neurons in the inferior vagal (nodose) ganglia from six pigs. Additional staining was performed for dopamine beta-hydroxylase (DBH), which is required for the production of norepinephrine (NE), to determine if these neurons are indeed sympathetic and capable of releasing NE. Analysis of stellate ganglia was also performed, given that these ganglia are known to provide sympathetic innervation to the heart and release NE in the myocardium. **Results:** While nearly all TH-expressing neurons in the stellate ganglia expressed DBH, confirming that they can produce or release NE, none of the TH-expressing neurons in the vagal ganglia expressed DBH, demonstrating that these are dopaminergic but not noradrenergic neurons. **Conclusions:** TH-expressing neurons in the vagal ganglia previously reported to be potentially “sympathetic” do not express DBH and are, therefore, not capable of synthesizing NE.

## 1. Introduction

The autonomic nervous system controls every aspect of cardiac function, including chronotropy, dromotropy, inotropy, and lusitropy. The cardiac autonomic nerves consist of afferent and efferent neurons whose cell bodies lie in the sympathetic and parasympathetic autonomic ganglia [1,2]. While cardiac sympathetic afferent signaling is transduced by neurons in the dorsal root ganglia, vagal sensory visceral activity is transduced by the neurons in the inferior vagal (nodose) ganglia [1,2]. Specifically, these ganglia contain cardiac sensory neurons that detect both mechanical and chemical cardiac stimuli and modulate vagal tone accordingly [3,4].

**Figure 1:**
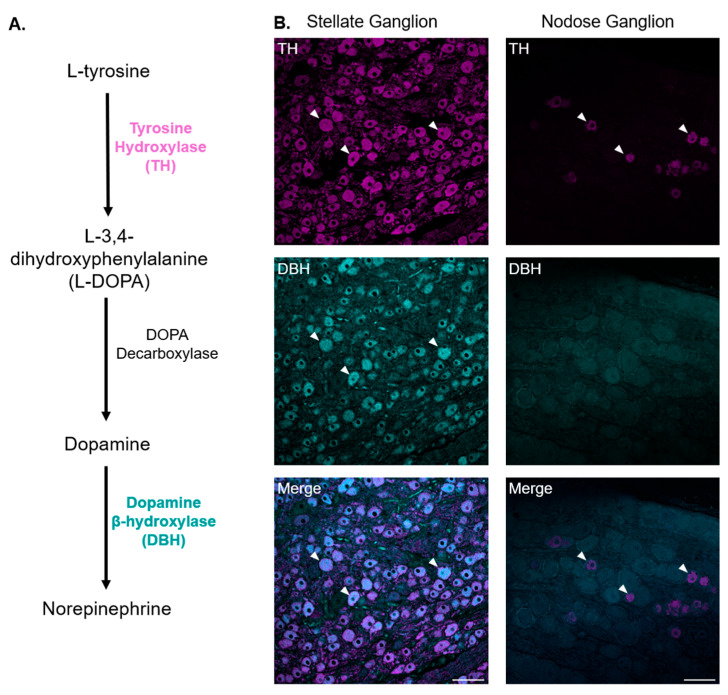
Catecholamine biosynthesis pathway and immunostaining for TH and DBH. (**A**) Diagram detailing the substrates and enzymes involved in the norepinephrine biosynthesis pathway. (**B**) Representative images of a section of a porcine stellate ganglion and nodose ganglion stained for TH and DBH. Arrowheads point to examples of neurons that express the specified marker of interest. Scale bars are 100 µm.

In the setting of structural heart disease, sympathoexcitation and parasympathetic dysfunction act in concert to contribute to the occurrence of ventricular arrhythmias and sudden cardiac death [5,6,7,8]. A healthy vagal tone acts as nature’s beta blocker, increasing the ventricular refractory period and calcium currents and reducing heart rate. Decreased vagal tone has been shown to increase the risk of ventricular arrhythmias and sudden cardiac death after myocardial infarction [9,10]. Neuromodulatory therapies that increase vagal tone, such as vagal nerve stimulation (VNS), are under investigation for the treatment of ventricular arrhythmias. VNS has been shown to increase the ventricular effective refractory period and action potential duration, decreasing the dispersion of repolarization and restitution, and reducing the occurrence of ischemia and scar-mediated ventricular arrhythmias in animal models [11,12,13,14,15,16,17,18]. However, clinical trials of VNS in heart failure have been met with mixed results [19,20,21,22]. Previous investigations have reported the presence of tyrosine hydroxylase (TH)-expressing neurons and fibers in the inferior vagal ganglia. TH is an enzyme involved in the biosynthesis of catecholamines and ultimately NE from tyrosine (Figure 1) [23]. It has been traditionally used as a marker for sympathetic nerves and neurons, including in the evaluation of cardiac peripheral sympathetic nervous system remodeling in response to pathologies, such as myocardial infarction and heart failure [24,25,26]. Thus, by demonstrating TH-immunoreactive neurons and fibers in the vagal ganglia and nerves, several studies have suggested that sympathetic activation with neuromodulation therapies, such as VNS, may occur and potentially counteract the desired outcome of increasing parasympathetic tone [27,28]. However, the role of TH is to convert tyrosine to L-3,4-dihydroxyphenylalanine (L-DOPA), and an additional enzyme, dopamine beta-hydroxylase (DBH), is required to convert dopamine to norepinephrine (NE) (Figure 1) [29,30]. Therefore, the presence of neuronal TH does not necessarily indicate the ability to synthesize NE.

The purpose of the current study was to more accurately characterize TH-expressing neurons in the inferior vagal (nodose) ganglia by staining for both TH and DBH in a swine model, a relevant large animal model that has been shown to undergo similar autonomic and cardiac remodeling after myocardial injury to humans [31,32]. We hypothesized that the neurons in these vagal ganglia possess dopaminergic but not noradrenergic characteristics. To further control for these experiments, we also stained the swine stellate ganglia for both TH and DBH, which would be expected to express both markers.

## 2. Methods

### 2.1. Ethical Approval

All animal experiments were approved by the University of California, Los Angeles Institutional Animal Care and Use Committee (ARC#2012-106, approval date 9 December 2024) and performed in accordance with the National Institutes of Health Guide for the Care and Use of Laboratory Animals.

### 2.2. Tissue Collection and Processing

Ganglia from healthy Yorkshire pigs (S&S Farms, Ramona, CA, USA, *n* = 6 males, 50–60 kg) were used for histological assessments. For ganglia where processing and staining yielded suboptimal results, tissue/slides were excluded from analysis. A total of 4 nodose (2 right and 2 left) and 4 stellate ganglia (2 right and 2 left) from these animals were used for analyses. Briefly, animals were sedated, intubated, and placed under general anesthesia with isoflurane (1.5–2.5% INH, MWI Animal Health, Boise, ID, USA), then switched to alpha-chloralose (MilliporeSigma, Burlington, MA, USA). Subsequently, bilateral neck cut-downs were performed, and the carotid sheaths were identified. Bilateral vagal trunks were then dissected and isolated in the carotid sheaths and followed cranially until the nodose ganglia were identified. The nodose ganglia were removed and fixed in 4% paraformaldehyde (Electron Microscopy Sciences, Hatfield, PA, USA) for 24 h. Subsequently, the tissue was washed and then placed in 70% ethanol (Thermo Fisher Scientific, Waltham, MA, USA) for storage at 4 °C until it was sectioned.

To collect the stellate ganglia, a sternotomy was performed under general anesthesia. The lungs were then gently retracted on each side, and the sympathetic chain was isolated behind the parietal pleura. The stellate ganglia were identified along the C7-T1 vertebrae, meticulously dissected, isolated, and removed. The stellate ganglia were then fixed in a similar fashion to the nodose ganglia. Prior to staining, the tissue was embedded in paraffin and sectioned (5 μm) longitudinally. Sections with the largest cross-sectional area for neurons from each ganglion were used for immunohistochemical analysis. At the end of the experiments, animals were euthanized.

### 2.3. Immunohistochemical Staining, Imaging, and Analysis

Tissue sections were deparaffinized through two washes of xylene (MilliporeSigma, Burlington, MA, USA) and one wash of xylene-ethanol solution. Then, the tissue sections were rehydrated with increasing dilutions of ethanol and distilled water solutions from 100% to 50% ethanol concentrations and finally washed with Tris-Buffered Saline (TBS, Bio-Rad, Hercules, CA, USA). Antigen retrieval was performed on the tissue sections using an EDTA buffer, pH 8.0 (Abcam, Cambridge, United Kingdom), at 95–100 °C for 30 min. Slides were blocked using 3% BSA-TBS (MilliporeSigma, Burlington, MA, USA) with 0.5% Triton X-100 (MilliporeSigma, Burlington, MA, USA) and 5% donkey serum (Jackson Immunoresearch, West Grove, PA, USA) for 1 h. Slides were subsequently incubated with primary antibodies overnight at 4 °C, then incubated with the appropriate affinity-purified F(ab’)_2_ fragment secondary antibodies (Table 1). Slides were then mounted using Vectashield Mounting Media (Vector Laboratories, Newark, CA, USA; H-1200). Finally, slides were imaged using a Zeiss LSM 880 with Airyscan confocal microscope (Zeiss, Oberkochen, Germany) at a magnification of 100× (10× objective and 10× eyepiece). Manual cell count was performed using the ImageJ Cell Counter plugin v1.54 (Bethesda, MD, USA). The percentage of TH- and DBH-expressing neurons was then quantified by normalizing to the total number of neuronal nuclei in each slide. Neurons were distinguished from other cell types, including glia, by their morphology, size, and nuclei.

### 2.4. Statistical Analysis

All data are presented as mean ± SEM. The percentage of neurons expressing TH or DBH was calculated for each cross-sectional area of each ganglion as a percentage of total neurons. Normality was assessed using the Shapiro–Wilk test. A 2-tailed unpaired Student’s *t*-test or Mann–Whitney U test was performed to determine differences between groups, depending on data distribution. *p* ≤ 0.05 was considered statistically significant. Analyses were performed using GraphPad Prism v10 (La Jolla, CA, USA). A power analysis was initially performed based on the assumption that more than 90% of stellate ganglia neurons would express DBH, while none of the TH-immunoreactive neurons in the nodose ganglia would be capable of synthesizing NE. This assumption was made based on the role of the stellate ganglion in providing sympathetic innervation to visceral organs, including the heart, and prior studies suggesting that TH-immunoreactive neurons in the nodose ganglia have a pseudo-unipolar morphology, suggesting that they are likely sensory neurons [33]. Hence, a total of 4 animals would be required for 90% power and a type I error rate (alpha) of 0.05.

## 3. Results

**Figure 2:**
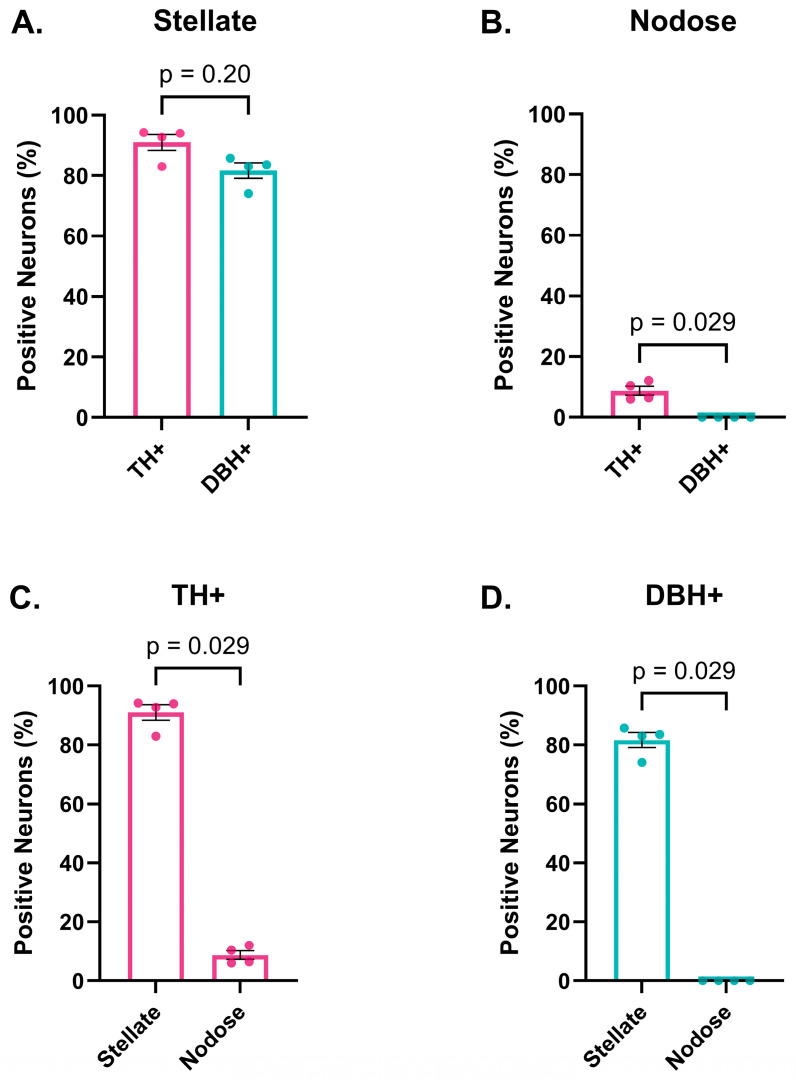
Comparison of TH- and DBH-immunoreactive neurons in the stellate ganglia and nodose ganglia. (**A**) No significant difference in the percentage of TH- and DBH-expressing neurons was found in the stellate ganglia (*n* = 4). (**B**) None of the TH-expressing neurons in the nodose ganglia were found to express DBH (*n* = 4). (**C**,**D**) As expected, the percentage of TH-expressing (TH+) neurons was significantly greater in the stellate as compared to nodose ganglia, as was the percentage of DBH-expressing (DBH+) neurons (*n* = 4 for both graphs). Data are shown as mean ± SEM; unpaired, Mann–Whitney U test. Each individual point on the bar graphs represents data from one animal.

As anticipated, the vast majority of neurons in the porcine stellate ganglia expressed TH (Figure 1 and Table 2). A comparison of TH vs. DBH expression demonstrated that the percentage of TH-expressing neurons was not significantly different from those that expressed DBH in the stellate ganglia (91.04% ± 2.69% for TH vs. 81.69% ± 2.58% for DBH, *p* = 0.20, Figure 2), suggesting that most stellate ganglia neurons are sympathetic and capable of producing NE. Conversely, the percentage of TH-expressing neurons was significantly greater than the percentage of DBH-positive neurons in the nodose ganglia (8.73% ± 1.47% for TH vs. 0.00% ± 0.00% for DBH, *p* = 0.029, Figure 2), as none of the TH-expressing neurons or any other neurons in the nodose ganglia were found to co-express DBH. This data suggested that TH-expressing neurons in the nodose ganglia are dopaminergic but not noradrenergic neurons (Figure 1 and Figure 2 and Table 2). As expected, the percentage of TH-positive neurons in the stellate ganglia was significantly greater than in the nodose ganglia (91.04% ± 2.69% in stellate ganglia vs. 8.73% ± 1.47% in nodose ganglia, *p* = 0.029, Figure 2).

## 4. Discussion

Cardiac function is tightly controlled by the autonomic nervous system to meet physiological demands. The autonomic nervous system has been traditionally divided into sympathetic and parasympathetic branches, and the activation of each branch has opposing effects on cardiac hemodynamic and electrophysiological parameters. The primary neurotransmitter of the sympathetic nervous system, NE, binds to adrenergic receptors on the myocardium and blood vessels and induces a signaling cascade that results in increased cardiac chronotropy, dromotropy, lusitropy, and inotropy. Conversely, postganglionic cholinergic neurons from the parasympathetic nervous system release acetylcholine, which binds to muscarinic receptors, resulting in reduced cardiac chronotropic, dromotropic, and inotropic hemodynamic parameters [5,34]. With structural heart disease, an increase in sympathetic activity and a decrease in parasympathetic function occur, and this autonomic remodeling is known to significantly increase the risk of ventricular arrhythmias and sudden cardiac death [1,5,34,35,36]. While the mechanisms of this pathological remodeling are still under active investigation, several potential underlying pathways have been identified. NE and its co-transmitter, neuropeptide Y, have been shown to act on adrenergic and Y2 receptors on myocardial parasympathetic nerve endings to further decrease acetylcholine release, promoting cardiac parasympathetic dysfunction [37,38]. Furthermore, sympathetic afferent nerves, activated with cardiac injury, act to inhibit central vagal drive, and blockade of nociceptive sympathetic afferent fibers has been shown to improve baroreflex function [39,40,41,42]. Finally, reduced vagal afferent signaling also appears to contribute to decreased vagal tone, as decreased nociceptive signaling of vagal sensory neurons has been shown to occur after chronic myocardial infarction [4,43]. As vagal afferent activation acts to increase efferent vagal tone, this sensory dysfunction could also contribute to parasympathetic dysfunction after myocardial infarction [2,44]. Therefore, the autonomic imbalance associated with cardiac disease appears to be due to sympathoexcitation and further inhibition of the central and peripheral parasympathetic nervous system by sympathetic afferent and efferent activation, as well as a reduction in parasympathetic afferent signaling.

To overcome the reduced vagal tone in the setting of structural heart disease, several neuromodulatory therapies, including VNS and baroreceptor activation, have been investigated. Specifically, VNS is under investigation as a neuromodulatory therapy to reduce atrial and ventricular arrhythmias after myocardial infarction and heart failure progression [11,12,19,45,46,47]. However, several studies have raised concerns about sympathetic activation with VNS, given the reported presence of TH-expressing vagal neurons and fibers in the nodose ganglia and vagal trunk reported in large animal models and humans [27,28]. Yet, these studies only evaluated the vagal nodose ganglia for neuronal TH immunoreactivity, without evaluating for DBH expression, which could help differentiate between noradrenergic vs. dopaminergic neurons. To understand whether neurons capable of synthesizing NE are present in the inferior vagal ganglia, we chose to employ a similar technique, immunohistochemistry, as in these prior studies. Our studies indeed confirmed the presence of TH-immunoreactive neurons in the nodose ganglia. However, none of these TH-expressing neurons co-expressed DBH. Therefore, these TH-expressing neurons do not appear to be capable of producing NE. On the other hand, nearly all the neurons in the stellate ganglia, which are known to provide noradrenergic sympathetic innervation to the heart, expressed both TH and DBH. It is important to note that we did not further confirm the presence of DBH in the nodose ganglia using quantitative polymerase chain reaction or Western blotting, which would have strengthened the results. Nevertheless, this study does provide insights into the phenotype and characteristics of the TH-expressing neurons in the vagal ganglia, demonstrating that they are dopaminergic and, therefore, unlikely to release NE upon vagal stimulation.

The current study did not perform functional evaluations to determine the role of these dopaminergic neurons/fibers in nodose vagal ganglia, and their function in visceral transduction is not entirely clear. A study by Katz and colleagues suggested that the TH-immunoreactive neurons of the rat nodose ganglion have morphological characteristics consistent with pseudo-unipolar sensory neurons, such as an initial axon glomerulus and a single, bifurcating neurite process, and therefore, are likely involved in visceral sensory transduction [33]. Lang et al. showed in the rat model that the esophagus and stomach are innervated by dopaminergic neurons in the nodose ganglia, suggesting that these neurons are likely involved in digestion and are separate from NPY-expressing neurons that are also known to be involved in gut sensory afferent signaling [48]. Dopaminergic neurons have also been shown to be involved in modulating synaptic plasticity in the central nervous system, but whether they play a similar role in vagal neurotransmission to the nucleus tractus solitarius remains unknown [49,50]. Additional studies on the physiological role of these neurons in visceral sensory neurotransmission are warranted.

## 5. Limitations

The current study used immunohistochemistry to evaluate for the presence of TH and DBH in the autonomic ganglia, without further confirmation with Western blotting or quantitative polymerase chain reaction. In addition, functional studies, including electrophysiological recordings or population-specific neural stimulations, were not performed to evaluate the physiological role of TH-immunoreactive neurons in the nodose ganglia. Future studies are warranted to evaluate the role of these dopaminergic neurons in visceral sensory transduction. Additionally, the study involved a small number of animals, which can reduce the reproducibility of the results. However, the data obtained for the lack of DBH in the nodose ganglia were consistent across all the animals evaluated. In accordance with our Institutional Animal Care and Use Committee protocol, the minimal number of animals required to obtain a statistically significant difference was used for the analysis, as pigs are a protected yet relevant species.

## 6. Conclusions

Using immunostaining data in a porcine model, this study demonstrates that TH-immunoreactive neurons on the nodose ganglia are dopaminergic, and not capable of synthesizing NE. This data has implications for neuromodulatory therapies that involve stimulation of the vagal trunk to achieve improve vagal tone.

## Figures and Tables

**Table 1:** Primary and secondary antibodies used for IHC.

Antibody	Host	Dilution	Vendor and Catalog #
Primary Antibody	Sheep Anti-TH	1:1000	MilliporeSigma, Burlington, MA, USA; ab1542
	Rabbit Anti-DBH	1:2000	Immunostar, Hudson, WI, USA; 22806
Secondary Antibody	Donkey Anti-Sheep IgG—AF647	1:200	Jackson Immunoresearch, West Grove, PA, USA; 713-605-147
	Donkey Anti-Rabbit IgG—AF555	1:200	Jackson Immunoresearch, West Grove, PA, USA; 711-565-152

**Table 2:** TH and DBH cell counts in stellate and nodose ganglia.

Ganglion	Animal Number	Total Neurons	TH+	% TH+	DBH+	% DBH+
Stellate Ganglia	Animal 1	2619	2470	94.31	2248	85.83
	Animal 2	2913	2740	94.06	2436	83.63
	Animal 3	1126	935	83.04	835	74.16
	Animal 4	2059	1910	92.76	1712	83.15
	Average	2179	2013	91.04	1807	81.69
Nodose Ganglia	Animal 3	982	118	12.02	0	0.00
	Animal 4	1116	116	10.39	0	0.00
	Animal 5	832	50	6.01	0	0.00
	Animal 6	1498	97	6.48	0	0.00
	Average	1107	95.25	8.73	0	0.00

+ indicates neurons expressing the specified marker.

## Data Availability

The quantified data supporting the conclusions of this articleare available to view under Supplementary Data. The raw data, including imaging files, will be made available by the authors upon request.

## Supplementary Materials

The following supporting information can be downloaded at: https://www.mdpi.com/article/10.3390/biomedicines13092126/s1, Supplementary Data.
